# Transduction of Adeno-Associated Virus Vectors Targeting Hair Cells and Supporting Cells in the Neonatal Mouse Cochlea

**DOI:** 10.3389/fncel.2019.00008

**Published:** 2019-01-24

**Authors:** Xi Gu, Renjie Chai, Luo Guo, Biao Dong, Wenyan Li, Yilai Shu, Xinsheng Huang, Huawei Li

**Affiliations:** ^1^Department of Otorhinolaryngology-Head and Neck Surgery, Zhongshan Hospital, Fudan University, Shanghai, China; ^2^Department of Otolaryngology-Head and Neck Surgery, The First Affiliated Hospital of Fujian Medical University, Fuzhou, China; ^3^Key Laboratory for Developmental Genes and Human Disease, Ministry of Education, Institute of Life Sciences, Southeast University, Nanjing, China; ^4^Co-innovation Center of Neuroregeneration, Nantong University, Nantong, China; ^5^Institute for Stem Cell and Regeneration, Chinese Academy of Sciences, Beijing, China; ^6^Jiangsu Province High-Tech Key Laboratory for Bio-Medical Research, Southeast University, Nanjing, China; ^7^ENT Institute and Otorhinolaryngology Department, Eye and ENT Hospital, State Key Laboratory of Medical Neurobiology, Fudan University, Shanghai, China; ^8^NHC Key Laboratory of Hearing Medicine, Fudan University, Shanghai, China; ^9^National Clinical Research Center for Geriatrics, State Key Laboratory of Biotherapy, West China Hospital, Sichuan University and Collaborative Innovation Center for Biotherapy, Chengdu, China; ^10^Institutes of Biomedical Sciences, Fudan University, Shanghai, China; ^11^Shanghai Engineering Research Center of Cochlear Implant, Shanghai, China; ^12^The Institutes of Brain Science and the Collaborative Innovation Center for Brain Science, Fudan University, Shanghai, China

**Keywords:** adeno-associated virus vectors, gene therapy, cochlea, hair cell, supporting cell, promoter

## Abstract

Adeno-associated virus (AAV) is the preferred vector for gene therapy of hereditary deafness, and different viral serotypes, promoters and transduction pathways can influence the targeting of AAV to different types of cells and the expression levels of numerous exogenous genes. To determine the transduction and expression patterns of AAV with different serotypes or promoters in hair cells and supporting cells in the neonatal mouse cochlea, we examined the expression of enhanced green fluorescent protein (eGFP) for five different types of AAV vectors [serotypes 2, 9, and Anc80L65 with promoter cytomegalovirus (CMV)-beta-Globin and serotypes 2 and 9 with promoter chicken beta-actin (CBA)] in *in vitro* cochlear explant cultures and we tested the transduction of AAV2/2-CBA, AAV2/9-CBA, and AAV2/Anc80L65-CMV by *in vivo* microinjection into the scala media of the cochlea. We found that each AAV vector had its own transduction and expression characteristics in hair cells and supporting cells in different regions of the cochlea. There was a tonotopic gradient for the *in vitro* transduction of AAV2/2-CBA, AAV2/9-CBA, AAV2/2-CMV, and AAV2/9-CMV in outer hair cells (OHCs), with more OHCs expressing eGFP at the base of the cochlea than at the apex. AAV2/2-CBA *in vitro* and AAV2/Anc80L65-CMV *in vivo* induced more supporting cells expressing eGFP at the apex than in the base. We found that AAV vectors with different promoters had different expression efficacies in hair cells and supporting cells of the auditory epithelium. The CMV-beta-Globin promoter could drive the expression of the delivered construct more efficiently in hair cells, while the CBA promoter was more efficient in supporting cells. The *in vitro* and *in vivo* experiments both demonstrated that AAV2/Anc80L65-CMV was a very promising vector for gene therapy of deafness because of its high transduction rates in hair cells. These results might be useful for selecting the appropriate vectors for gene delivery into different types of inner ear cells and thus improving the effectiveness of gene therapy.

## Introduction

Noise, ototoxic drugs, infections, autoimmune diseases, and hereditary factors can cause loss of hair cells (HCs) and/or functional deficiency of HCs and supporting cells (SCs). Such changes are usually irreversible and account for a large proportion of all incidences of sensorineural hearing loss. The treatment for hereditary deafness caused by hundreds of different gene mutations has traditionally been hearing aids or cochlear implants, but these rarely achieve the desired effect. Gene therapy is therefore a very promising approach to restoring hearing in patients with hereditary deafness (Akil et al., 2012; Lentz et al., 2013; Yu et al., 2014; Askew et al., 2015; Zuris et al., 2015; Chien et al., 2016; Gao et al., 2018).

Adeno-associated virus (AAV) is the preferred vector for gene therapy in the treatment of sensorineural hearing loss (Li Duan et al., 2002; Lustig and Akil, 2012). Different serotypes, promoters, and transduction pathways can influence the targeting of AAV to different types of cells (Sacheli et al., 2013); for example, AAV2/2 can transduce HCs and SCs *in vitro* (Stone et al., 2005), while introduction of AAV2/2 into the inner ear through the round window can only transduce the spiral limbus, spiral ganglion cells, and the spiral ligament (Jero et al., 2001; Luebke et al., 2001a; Li Duan et al., 2002). Morphologically, gene expression has been shown to be more abundant after injection into the scala media compared to injection into the scala tympani (Shibata et al., 2009), and it has been shown that delivery of AAV1-Kcnq1-GFP into the inner ear through the scala media can induce the expression of target genes in marginal cells of the stria vascularis (Chang et al., 2015). Yu et al. (2014) inoculated modified AAV vectors into the scala media of early postnatal conditional *Gjb2* knockout mice to drive exogenous connexin26 expression. They found extensive virally expressed connexin26 in cells lining the scala media, and the intercellular gap junction network was re-established in the organ of Corti of the mutant mouse cochlea, although auditory brainstem responses (ABRs) did not show significant hearing improvement. Akil et al. (2012) reported the successful restoration of hearing in the *Vglut3* knockout mouse using AAV-mediated gene delivery. They found that cochlear delivery of Vglut3 using AAV1 led to transgene expression only in inner hair cells (IHCs), and within 2 weeks of AAV1-Vglut3 delivery the click ABR thresholds had almost normalized. These findings indicate the successful restoration of hearing by gene replacement in mice, which is an important step toward gene therapy for human deafness.

Deafness results from damage to many different cell types in the inner ear. To cure deafness by restoring the structure and function of the damaged cells, we need more knowledge about the transduction characteristics of AAV vectors so as to choose the most appropriate AAV vectors for specific and accurate transgene expression. To clarify the targeting and expression features of AAV vectors with different serotypes or promoters, we investigated the transduction efficiencies of five different AAV vectors [serotypes 2, 9, and Anc80L65 with the cytomegalovirus (CMV)-beta-Globin promoter and serotypes 2 and 9 with the chicken beta-actin (CBA) promoter] in HCs and SCs in the apical, middle, and basal turns of the cochlea. We also chose AAV2/2-CBA, AAV2/9-CBA, and AAV2/Anc80L65-CMV for our *in vivo* experiments due to their high expression efficacy in SCs *in vitro*. While there have been many studies (Liu et al., 2005; Stone et al., 2005; Bedrosian et al., 2006; Ballana et al., 2008; Konishi et al., 2008; Kilpatrick et al., 2011; Shu et al., 2016; Landegger et al., 2017) comparing the transduction efficiencies of different AAV serotypes in inner ear cells, few studies (Liu et al., 2007; Luebke et al., 2009) have focused on the difference in expression efficacy between promoters. Luebke et al. (2009) found that AAV1/2 using astrocyte-specific promoters [the glial fibrillary acid protein (GFAP) and the brain lipid-binding protein (BLBP)] had the potential to drive SC-specific expression in the adult guinea pig cochlea, but the injection of AAV1/2-GFAP/BLBP-eGFP caused the loss of outer hair cells (OHCs). In their experiments, they did not compare the expression efficacy between the two promoters in SCs. Liu et al. (2007) evaluated the expression of enhanced green fluorescent protein (eGFP) driven by six different promoters, including the CMV IE enhancer and CBA promoter (CAG), the CMV promoter, the neuron-specific enolase promoter, the myosin 7A (Myo7a) promoter, the elongation factor 1α promoter, and the Rous sarcoma virus promoter, but the level of eGFP expression was graded by fluorescent intensity rather than presented as a specific number of eGFP positive (eGFP^+^) cells. To date, there have been no reports comparing the expression efficacy of AAV vectors with different promoters in SCs. Few studies have directly compared the performance of viral vectors in titer and promoter-matched and controlled conditions as has been done in the present work. In this work, we studied the transduction patterns of AAV vectors with different serotypes, and we present detailed data on transduction efficiencies in different regions of the cochlea. We found that AAV vectors with different promoters (CMV-beta-Globin or CBA) had different expression efficacies in HCs and SCs of the auditory epithelium. We conclude that CMV-beta-Globin is a superior promoter for driving expression of the delivered construct in HCs, while the CBA promoter is superior at driving expression in SCs.

## Materials and Methods

### Animals

We used C57BL/6J wild type mice to examine the transduction efficiency of different types of AAV vectors *in vitro*, and we used ICR wild type mice for the *in vivo* experiments. All animal experiments were approved by the Institutional Animal Care and Use Committee of Fudan University.

### Viral Vectors

We tested the transduction efficiency of five different types of AAV vectors that mediated the expression of eGFP. Three of the AAV vectors had the CMV-beta-Globin promoter and the Woodchuck hepatitis virus post-transcriptional regulatory element cassette (original titer given in parentheses), namely AAV2/2-CMV-beta-Globin-eGFP [1 × 10^13^ viral genomes (VG) per ml], AAV2/9-CMV-beta-Globin-eGFP (1 × 10^13^ VG/ml) and AAV2/Anc80L65-CMV-beta-Globin-eGFP (2.08 × 10^12^ VG/ml), and these were purchased from the Biolink Company (Shanghai, China). The other two AAV vectors had the CBA promoter, namely AAV2/2-CBA-eGFP (1 × 10^13^ VG/ml) and AAV2/9-CBA-eGFP (1 × 10^13^ VG/ml), and these were kindly given as gifts from Professor Biao Dong of Sichuan University. The AAV2/Anc80L65 plasmid reagents are available through http://www.addgene.com. The CMV-beta-Globin promoter is comprised of a CMV immediate early enhancer and the CMV promoter itself. The intron part of the rabbit beta-Globin gene is in the promoter. The origin of the CBA promoter is from a well-characterized plasmid pscAAV-CBA-eGFP which was used for the packaging of a self-complementary AAV vector. The part we took is the CBA promoter and an intron just behind it which was widely used to enhance the expression of the gene of interest. The eGFP expression was analyzed 7 days after virus incubation *in vitro* and 1 month after virus injection *in vivo*.

All the AAV vectors were generated utilizing the triple-plasmid co-transfection method as previously described (Wang et al., 2014). The AAV vectors from Sichuan University were purified by two rounds of cesium chloride (CsCl) gradient ultracentrifugations (Wang et al., 2014), and the AAV vectors from the Biolink Company were purified using the discontinuous iodixanol density gradient centrifugation procedure (Grieger et al., 2006). Virus titers were measured by quantitative polymerase chain reaction (qPCR) as previously described (Wang et al., 2016). The previous results showed that the full particles in the vectors purified using the CsCl gradient centrifugation procedure were >90% by electronic microscopy (Wang et al., 2016), while the full particles in the vectors purified using the iodixanol gradient centrifugation procedure were >80% (Grieger et al., 2006).

Virus purity of the AAV vectors from Sichuan University was examined by silver staining after separation using a sodium dodecyl sulfate polyacrylamide gel electrophoresis (SDS-PAGE) gel and the purity of VPs of the vectors was >90%. For the purity test of the AAV vectors from the Biolink Company, the protein samples were prestained by Band-Now Pre-Staining Protein Sample Treatment Buffer (Sangon Biotech) during sample treatment prior to electrophoresis and the result of protein purity could be directly observed after SDS-PAGE under an UV transilluminator. The results showed that the purity of vectors from the Biolink Company was >95%.

We obtained two different lots of virus for each AAV vector. To validate the quality and stability of virus, we tested the transduction efficiency of two lots of virus in cochlear explant cultures with the same experimental conditions and compared the transduction efficiencies of AAV vectors in HCs between the two different lots.

### *In vitro* Cochlear Explant Cultures

After being dissected in pre-chilled phosphate buffered saline (PBS, Hyclone), the cochleae of postnatal day 1 (P1) C57BL/6J mice were cultured in DMEM/F12 (Hyclone) with 1% N2 supplement (Life Technologies), 2% B27 supplement (Life Technologies), and 50 μg/ml ampicillin (Sigma) at 37°C with 5% CO_2_. The virus was applied after 2 h in culture, and all of the explants were cultured in the nutrient solution with AAV vectors for 48 h. The medium was then changed to a DMEM/F12 mixture without virus, and the explants were cultured for another 5 days. During the culture, the medium was changed every other day.

### *In vivo* AAV Microinjections

P2–3 ICR mice were used for AAV2/2-CBA, AAV2/9-CBA and AAV2/Anc80L65-CMV-beta-Globin injection. Mice were anesthetized with low temperature exposure by placing them on ice, and a skin incision was made behind the right ear of the mouse to expose the otic bulla and the stapedial artery. Glass micropipettes (WPI, Sarasota, FL) held by a Nanoliter Microinjection System (WPI) were used to penetrate into the scala media through the soft cochlear lateral wall. The total injection volume was 0.2 μl per cochlea, and the injection was controlled by a micromanipulator at a speed of 3 nl/s (Shu et al., 2016). The mice were sacrificed at 1 month after AAV injection.

### Hearing Tests After AAV Injection

All injected animals survived the surgery. The ABR test was used to measure hearing 1 month after virus injection, and the un-injected ears were used as controls. Tone burst sound stimuli were presented at 4, 8, 16, and 24 kHz to test frequency-specific hearing thresholds as previously described (Huang et al., 2013). The ABR thresholds were analyzed blinded.

### Immunohistochemistry

After being cultured for a total of 7 days *in vitro*, the cochleae were fixed with 4% (wt/vol) paraformaldehyde (Sigma) for 30 min at room temperature. All samples were then permeabilized and blocked in PBS containing 1% Triton X-100 (1% PBS-T) and 10% donkey serum for 1 h at room temperature. All primary and secondary antibodies were diluted in 1% PBS-T. To observe the eGFP expression in HCs and SCs of the cochlear sensory epithelium, we used rabbit anti-Myo7a (1:600 dilution, Proteus BioSciences) and goat anti-Sox2 (1:300 dilution, Santa Cruz) primary antibodies to label HCs and SCs, respectively. To compare the transduction rates between different samples in the same fluorescence condition, all of the *in vitro* samples were analyzed by the cellular eGFP fluorescence itself, while the eGFP fluorescence of *in vivo* samples was enhanced by immunolabeling with chicken anti-eGFP primary antibody (1:1,000 dilution, Abcam). Appropriate Alexa-conjugated secondary antibodies were used for detection, and DAPI was used to label the nuclei (1:1,000 dilution, Sigma).

Cochleae were dissected from the temporal bones at 1 month after AAV2/2-CBA, AAV2/9-CBA, and AAV2/Anc80L65-CMV injection *in vivo*, and the samples were fixed with 4% paraformaldehyde for 60 min at room temperature. After rinsing with PBS three times, we soaked all of the cochleae in 10% EDTA for 3 days for decalcification. The entire basilar membrane was divided into three pieces of equal length designated the basal, middle, and apical turns of the cochlea. Subsequent staining steps followed the same procedures as described above.

### Cell Counting and Statistics

A Leica TCS SP8 laser scanning confocal microscope was used to observe the immunohistochemical staining and to collect fluorescent Z-stack images. We used a 40× objective for microscopy, and the z-step size was 1 μm. The maximum intensity projections of optical confocal sections are displayed in the figures. For cell counting, the numbers of Myo7a^+^ HCs, Sox2^+^ SCs outside of IHCs, Myo7a^+^/eGFP^+^ cells and Sox2^+^/eGFP^+^ cells were counted in every 200 μm region of the apical, middle, and basal turns of the cochlea using ImageJ software. For *in vitro* experiments, five 200 μm regions were counted to derive a value for each “Apex,” “Mid,” and “Base” region when calculating the transduction efficiency of AAV vectors in HCs of the three regions, respectively. Four 200 μm regions were counted to calculate the transduction efficiency of AAV vectors in SCs of the three regions, respectively. In some cases, the average transduction efficiency of AAV vectors in SCs of the whole cochlea was calculated by averaging the transduction efficiencies of the three regions. For *in vivo* experiments, at least three samples in each group from three independent experiments were collected. The transduction efficiencies of AAV vectors targeting HCs or SCs were calculated as the ratio of Myo7a^+^/eGFP^+^ cells to Myo7a^+^ cells or the ratio of Sox2^+^/eGFP^+^ cells to Sox2^+^ cells, respectively. The results are presented as the mean ± standard deviation, and all statistical analyses were performed with GraphPad Prism 6.0. In the case of abnormal distribution, a non-parametric statistical test (Mann–Whitney *U* test) was applied to analyze the data. In the case of normal distribution and equal variance, unpaired two-tailed Student’s *t*-test was applied to determine statistical significance. Unpaired *t*-test with Welch’s correction was performed if the data conformed to a normal distribution but equal variance could not be assumed. When multiple comparison tests were applied to compare the transduction efficiencies of AAV vectors between different groups in the three regions of the cochlear, *P* < 0.0167 was considered statistically significant after Bonferroni correction. In other cases, *P* < 0.05 was considered statistically significant.

## Results

### The Observation of the Culture Explants of the Neonatal Mouse Cochlea After AAV Incubation

After being cultured in the DMEM/F12 medium containing AAV vectors for 48 h, a small number of fibroblasts around the basilar membrane expressed eGFP. With longer culture time, the expression of eGFP in the sensory epithelium of the cochlea gradually increased, and the cells of the basal turn usually expressed eGFP before the other turns. On the fourth day of culture, the IHCs and OHCs presented different fluorescence intensities in different AAV groups.

### Results of Validations With Multiple Lots of Each AAV Vector

The transduction efficiencies of AAV2/2-CBA, AAV2/9-CBA, AAV2/2-CMV, AAV2/9-CMV, and AAV2/Anc80L65-CMV with two different lots were presented in Supplementary Table 1. The transduction efficiencies in IHCs and OHCs of the apical, middle, and basal turns of the cochlea between two different lots were not statistically significantly different which indicated that the effectiveness of virus from both facilities were stable.

### The Transduction Efficiency of AAV Vectors Targeting HCs With Different Working Titers

When the working titer was increased from 1 × 10^11^ VG/ml to 1 × 10^12^ VG/ml, the transduction efficiency of AAV2/2-CBA in IHCs and OHCs did not increase statistically significantly in the apical, middle, or basal turn of the cochlea. The percentage of eGFP^+^ IHCs was no more than 10% throughout the cochlea on average at a titer of 1 × 10^12^ VG/ml. This suggests that even when the viral titer was very high, AAV2/2 with the CBA promoter still could not efficiently transduce IHCs (Table 1 and Supplementary Figure 1). However, the transduction efficiency of AAV2/9-CBA in IHCs throughout the cochlea and in OHCs of the apical and middle turns increased statistically significantly as the titer increased. The transduction efficiency of AAV2/9-CBA was concentration-dependent in the IHCs and OHCs and there was a greater increase in transduction efficiency in IHCs (∼6-fold) compared to OHCs (∼2-fold) (Table 1 and Supplementary Figure 1).

**Table 1:** The transduction efficiencies of AAV2/2-CBA and AAV2/9-CBA in IHCs, OHCs, and SCs in the conditions of different viral titers.

Serotype	Cell type	Viral titer (VG/ml)	Apex (%)	Mid (%)	Base (%)
AAV2/2	IHCs	1 × 10^11^	6.0 ± 3.2	3.9 ± 2.5	11.7 ± 12.7
		1 × 10^12^	7.3 ± 5.8	9.1 ± 10.4	3.6 ± 2.1
	OHCs	1 × 10^11^	35.1 ± 3.3	47.8 ± 16.4	68.2 ± 17.7
		1 × 10^12^	56.2 ± 18.0	62.1 ± 21.7	75.2 ± 9.2
	SCs	1 × 10^11^	35.8 ± 10.6	6.9 ± 9.1	4.2 ± 4.1
		5 × 10^11^	47.1 ± 7.4	32.5 ± 30.6	24.6 ± 24.7
		1 × 10^12^	61.4 ± 21.9	40.8 ± 38.3	7.6 ± 1.4
AAV2/9	IHCs	1 × 10^11^	6.9 ± 2.9	4.2 ± 0.9	6.6 ± 1.7
		1 × 10^12^	39.0 ± 17.4	41.6 ± 11.0	40.1 ± 14.9
	OHCs	1 × 10^11^	4.5 ± 1.1	3.0 ± 1.9	26.2 ± 5.9
		1 × 10^12^	25.9 ± 7.0	30.5 ± 12.2	17.9 ± 6.2
	SCs	1 × 10^11^	4.3 ± 5.1	0	5.5 ± 11.1
		5 × 10^11^	10.2 ± 11.8	19.1 ± 16.4	4.3 ± 5.2
		1 × 10^12^	65.1 ± 20.6	53.6 ± 15.1	39.1 ± 11.8

*The values were represented as mean ± standard deviation (n = 5 for HCs; n = 4 for SCs).*

As the titer increased from 0.5 × 10^11^ VG/ml to 1 × 10^11^ VG/ml, the transduction efficiency of AAV2/2-CMV in IHCs in the basal turn of the cochlea increased statistically significantly (∼2-fold). However, the percentage of eGFP^+^ OHCs did not increase statistically significantly. It is possible that in the case of the lower concentration AAV2/2-CMV preferentially transduced OHCs, and as the concentration increased more IHCs could be transduced (Table 2 and Supplementary Figure 2). The transduction efficiency of AAV2/9-CMV in IHCs and OHCs did not increase statistically significantly as the titer increased (Table 2 and Supplementary Figure 2). There was a tonotopic gradient for the transduction efficiency of AAV2/2-CBA, AAV2/9-CBA, AAV2/9-CMV at a titer of 1 × 10^11^ VG/ml, and AAV2/2-CMV at a titer of 0.5 × 10^11^ VG/ml, with more OHCs expressing eGFP at the base of the cochlea than at the apex (Supplementary Figure 5).

**Table 2:** The transduction efficiencies of AAV2/2, AAV2/9, and AAV2/Anc80L65 with the CMV-beta-Globin promoter in IHCs, OHCs, and SCs in the conditions of different viral titers.

Serotype	Cell type	Viral titer (VG/ml)	Apex (%)	Mid (%)	Base (%)
AAV2/2	IHCs	0.5 × 10^11^	17.9 ± 14.0	20.0 ± 5.0	35.1 ± 16.6
		1 × 10^11^	35.9 ± 24.3	45.5 ± 18.8	67.0 ± 6.5
	OHCs	0.5 × 10^11^	56.2 ± 11.9	65.8 ± 14.3	81.2 ± 6.2
		1 × 10^11^	68.3 ± 19.8	63.8 ± 22.8	76.0 ± 21.6
	SCs	1 × 10^11^	14.8 ± 19.6	0	5.0 ± 10.0
		5 × 10^11^	18.6 ± 7.4	19.7 ± 13.7	36.4 ± 5.9
		1 × 10^12^	7.3 ± 8.6	5.5 ± 6.7	21.2 ± 15.0
AAV2/9	IHCs	0.5 × 10^11^	43.7 ± 20.6	36.0 ± 25.2	52.1 ± 21.2
		1 × 10^11^	47.9 ± 19.2	58.8 ± 20.0	73.6 ± 6.5
	OHCs	0.5 × 10^11^	7.1 ± 9.8	11.4 ± 10.8	17.6 ± 17.9
		1 × 10^11^	12.1 ± 11.7	30.1 ± 12.6	53.5 ± 28.1
	SCs	1 × 10^11^	0	0	0
		5 × 10^11^	5.5 ± 5.8	14.4 ± 17.1	5.6 ± 3.7
		1 × 10^12^	3.5 ± 5.7	0	17.4 ± 15.5
AAV2/Anc80L65	IHCs	0.5 × 10^11^	28.2 ± 15.5	25.6 ± 11.5	26.0 ± 12.7
		1 × 10^11^	76.7 ± 11.8	82.5 ± 6.4	83.8 ± 8.8
		2 × 10^11^	97.8 ± 3.1	97.7 ± 3.4	98.3 ± 2.3
	OHCs	0.5 × 10^11^	24.3 ± 15.3	27.9 ± 13.0	33.6 ± 12.4
		1 × 10^11^	68.9 ± 8.4	75.8 ± 6.6	81.6 ± 8.0
		2 × 10^11^	93.1 ± 5.9	95.3 ± 1.2	98.2 ± 1.9
	SCs	1 × 10^11^	0	7.1 ± 10.1	0
		2 × 10^11^	45.3 ± 19.7	48.4 ± 25.8	15.1 ± 3.5
		5 × 10^11^	7.0 ± 14.1	2.6 ± 3.7	21.4 ± 12.3

*The values were represented as mean ± standard deviation (n = 5 for HCs; n = 4 for SCs).*

We tested three virus titers, including 0.5 × 10^11^ VG/ml, 1 × 10^11^ VG/ml, and 2 × 10^11^ VG/ml, for the transduction of AAV2/Anc80L65-CMV. The transduction efficiencies of AAV2/Anc80L65-CMV in IHCs and OHCs were clearly concentration-dependent. With the concentration elevated within a narrow range, the percentage of both eGFP^+^ IHCs and eGFP^+^ OHCs increased statistically significantly. The transduction efficiencies in IHCs of the apical, middle, and basal turns were 97.8 ± 3.1%, 97.7 ± 3.4%, and 98.3 ± 2.3%, respectively, and the transduction efficiencies in OHCs of the apical, middle and basal turns were 93.1 ± 5.9%, 95.3 ± 1.2%, and 98.2 ± 1.9%, respectively, at a titer of 2 × 10^11^ VG/ml. Thus, the viral transduction rates of AAV2/Anc80L65-CMV in IHCs and OHCs were consistently high along the base-to-apex axis (Table 2, Supplementary Figure 2, and Figure 3).

### Comparison of the Transduction Efficiencies Targeting HCs Between Different Serotypes of AAV Vectors With the Same Promoters

**FIGURE 1:**
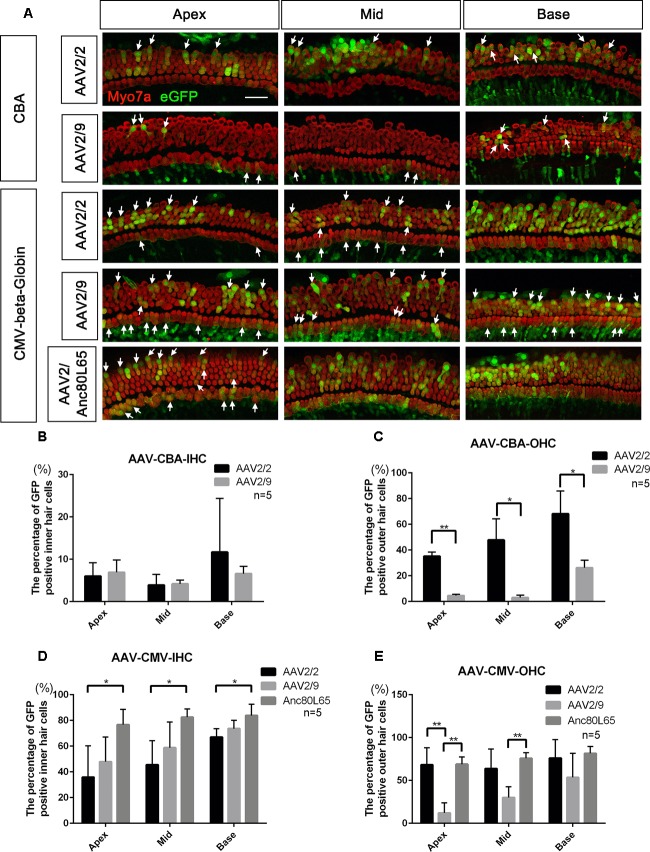
Comparison of the *in vitro* transduction efficiencies in targeting HCs between different serotypes of AAV with the same promoters. **(A)** Representative confocal images of transduction of five AAV vectors (serotypes 2 and 9 with the CBA promoter and serotypes 2, 9, and Anc80L65 with the CMV-beta-Globin promoter) in HCs of the apical, middle, and basal turns of the cochlea. The arrows indicate the eGFP^+^ HCs. **(B)** Comparison of the transduction efficiencies between AAV serotypes 2 and 9 with the CBA promoter in targeting IHCs at a titer of 1 × 10^11^ VG/ml. **(C)** Comparison of the transduction efficiencies between AAV2/2-CBA and AAV2/9-CBA in targeting OHCs at a titer of 1 × 10^11^ VG/ml. AAV2/2-CBA clearly transduced more OHCs than AAV2/9-CBA in the apical, middle, and basal turns of the cochlea. **(D)** Comparison of the transduction efficiencies between serotypes Anc80L65, 2, and 9 with the same CMV-beta-Globin promoter in targeting IHCs at a titer of 1 × 10^11^ VG/ml. Overall, the transduction efficiency of AAV2/Anc80L65 was statistically significantly greater than the transduction efficiency of AAV2/2. There was no statistically significant difference in the percentages of eGFP^+^ IHCs between AAV2/2 and AAV2/9. **(E)** Comparison of transduction efficiencies between the three AAV vectors with the CMV-beta-Globin promoter when targeting OHCs at a titer of 1 × 10^11^ VG/ml. AAV2/Anc80L65 transduced more OHCs in the apical and middle turns than AAV2/9, while AAV2/2 transduced more OHCs in the apical turn than AAV2/9. ^∗^*p* < 0.0167, ^∗∗^*p* < 0.0033, *n* = 5. Scale bar: 20 μm.

When the promoter was CBA, the differences in transduction efficiencies in IHCs between AAV2/2 and AAV2/9 were not statistically significant, and both of the transduction efficiencies were very low (<10%). However, AAV2/2-CBA had higher transduction efficiency than AAV2/9-CBA in OHCs in the apical (35.1 ± 3.3% vs. 4.5 ± 1.1%), middle (47.8 ± 16.4% vs. 3.0 ± 1.9%), and basal (68.2 ± 17.7% vs. 26.2 ± 5.9%) turns of the cochlea (Table 1 and Figure 1). With the CMV-beta-Globin promoter, AAV2/Anc80L65 had the overall highest transduction efficiency among the three serotypes in IHCs, while AAV2/9 had the lowest transduction efficiency in OHCs (Table 2 and Figure 1).

### Comparison of the Expression Efficacy in HCs Between AAV Vectors With Different Promoters

**FIGURE 2:**
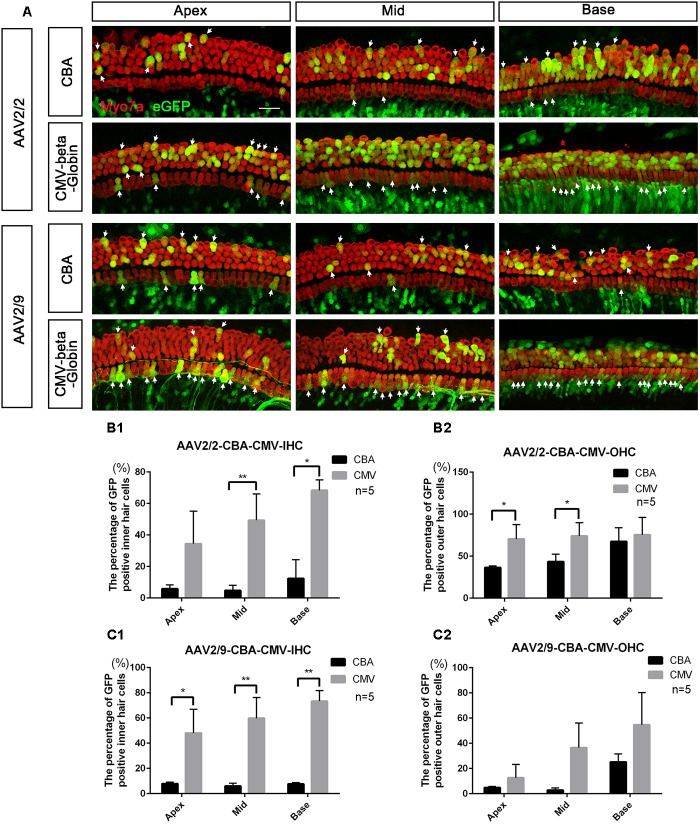
Comparison of the *in vitro* expression efficacy in HCs between AAV vectors with different promoters. **(A)** Representative confocal images of transduction of AAV2/2-CBA, AAV2/2-CMV-beta-Globin, AAV2/9-CBA, and AAV2/9-CMV-beta-Globin in HCs of the apical, middle, and basal turns of the cochlea. The arrows indicate the eGFP^+^ HCs. **(B1)** Comparison of the expression efficiencies between AAV2/2-CBA and AAV2/2-CMV-beta-Globin in IHCs at a titer of 1 × 10^11^ VG/ml. **(B2)** Comparison of the expression efficiencies between AAV2/2-CBA and AAV2/2-CMV-beta-Globin in OHCs at a titer of 1 × 10^11^ VG/ml. AAV2/2-CMV-beta-Globin induced more IHCs in the middle and basal turns and more OHCs in the apical and middle turns of the cochlea to express eGFP than AAV2/2-CBA. **(C1)** Comparison of the expression efficiencies between AAV2/9-CBA and AAV2/9-CMV-beta-Globin in IHCs at a titer of 1 × 10^11^ VG/ml. **(C2)** Comparison of the expression efficiencies between AAV2/9-CBA and AAV2/9-CMV-beta-Globin in OHCs at a titer of 1 × 10^11^ VG/ml. AAV2/9-CMV-beta-Globin induced more IHCs throughout the cochlea to express eGFP than AAV2/9-CBA. ^∗^*p* < 0.0167, ^∗∗^*p* < 0.0033, *n* = 5. Scale bar: 20 μm.

When the working titer of virus was 1 × 10^11^ VG/ml, the expression efficacy of AAV2/2-CMV in the IHCs and OHCs was statistically significantly greater than that of AAV2/2-CBA, with about a 6-fold increase in IHCs and about a 1.5-fold increase in OHCs (Table 3 and Figure 2). Similarly, AAV2/9-CMV had higher expression efficacy than AAV2/9-CBA in HCs, with about an 8-fold increase in IHCs and about a 3-fold increase in OHCs (Table 4 and Figure 2). This indicates that the CMV-beta-Globin promoter is more efficient than the CBA promoter when driving target gene expression in cochlear HCs.

**Table 3:** Comparison of the expression efficiencies in IHCs and OHCs between AAV2/2-CBA and AAV2/2-CMV-beta-Globin when the viral titer was 1 × 10^11^ VG/ml.

Promoter	Cell type	Apex (%)	Mid (%)	Base (%)
CBA	IHCs	5.8 ± 2.5	4.8 ± 3.3	12.4 ± 11.9
	OHCs	36.6 ± 1.8	43.6 ± 8.7	67.6 ± 16.2
CMV-beta-Globin	IHCs	34.4 ± 20.7	49.4 ± 16.7	68.4 ± 6.7
	OHCs	70.4 ± 17.3	74.0 ± 15.8	75.4 ± 20.6

*The values were represented as mean ± standard deviation (n = 5).*

**Table 4:** Comparison of the expression efficiencies in IHCs and OHCs between AAV2/9-CBA and AAV2/9-CMV-beta-Globin when the viral titer was 1 × 10^11^ VG/ml.

Promoter	Cell type	Apex (%)	Mid (%)	Base (%)
CBA	IHCs	7.9 ± 1.2	6.0 ± 2.30	7.6 ± 1.2
	OHCs	4.9 ± 0.9	2.9 ± 1.7	25.2 ± 6.4
CMV-beta-Globin	IHCs	48.0 ± 18.9	59.8 ± 16.4	73.4 ± 8.3
	OHCs	12.6 ± 10.7	36.5 ± 19.5	54.7 ± 25.6

*The values were represented as mean ± standard deviation (n = 5).*

### The Transduction Efficiency of AAV Vectors Targeting SCs With Different Working Titers

As the virus titer was increased by 5-fold or 10-fold, the transduction efficiency of AAV2/2-CBA in SCs in the apical, middle, and basal turns did not increase statistically significantly. When the working titer of virus was increased from 5 × 10^11^ VG/ml to 1 × 10^12^ VG/ml, the average transduction efficiency of AAV2/9-CBA in SCs of the whole cochlea increased statistically significantly (11.20 ± 2.88% vs. 52.58 ± 12.02%, *p* = 0.0048). Unlike the tonotopic gradient in HCs, AAV2/2-CBA induced more SCs expressing eGFP at the apex of the cochlea than in the base at a titer of 1 × 10^11^ VG/ml (Table 1 and Supplementary Figures 3, 5).

As the virus titer was increased, the transduction efficiencies of AAV2/2-CMV and AAV2/9-CMV in SCs in the apical, middle, and basal turns did not increase significantly (Table 2 and Supplementary Figure 3). When compared in the three regions of the cochlea, respectively, the transduction efficiencies of AAV2/Anc80L65-CMV in SCs did not increase statistically significantly with the working titer increased. The average transduction efficiencies of AAV2/Anc80L65-CMV in SCs of the whole cochlea increased significantly (2.35 ± 3.36% vs. 36.24 ± 13.00%, *p* = 0.0023) with the working titer increased from 1 × 10^11^ VG/ml to 2 × 10^11^ VG/ml. Surprisingly, the average transduction efficiencies in SCs of the whole cochlea decreased instead of increasing (36.24 ± 13.00% vs. 10.33 ± 4.93%, *p* = 0.0098) when the titer was increased from 2 × 10^11^ VG/ml to 5 × 10^11^ VG/ml (Table 2 and Supplementary Figure 3).

### Comparison of Transduction Efficiencies Targeting SCs of the Cochlea Between Different Serotypes of AAV Vectors With the Same Promoters

The difference in transduction efficiencies in SCs of the apical, middle, and basal turns between AAV2/2-CBA and AAV2/9-CBA was not statistically significant at a titer of 5 × 10^11^ VG/ml (Table 1 and Supplementary Figure 4).

**FIGURE 3:**
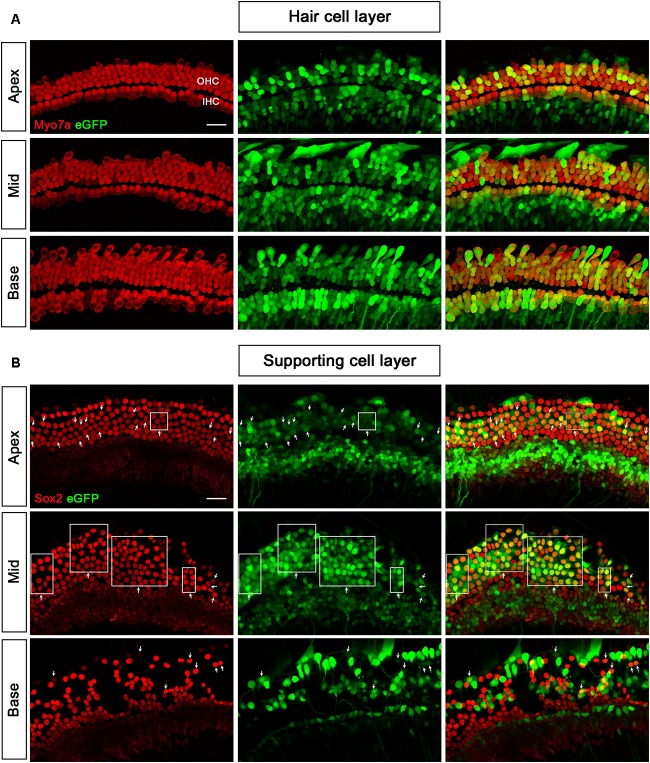
Transduction of AAV2/Anc80L65-CMV-beta-Globin in HCs and SCs at a titer of 2 × 10^11^ VG/ml. **(A)** In the HC layer, the arrows indicate the Myo7a^+^/eGFP^+^ HCs. **(B)** In the SC layer, the arrows indicate the Sox2^+^/eGFP^+^ SCs. The square frames indicate the regions of SCs expressing eGFP.

When the working titer of AAV2/2-CMV and AAV2/9-CMV was 5 × 10^11^ VG/ml and the working titer of AAV2/Anc80L65-CMV was 2 × 10^11^ VG/ml, the transduction efficiencies of the three serotypes in the basal turn ranked from high to low were AAV2/2 > AAV2/Anc80L65 > AAV2/9. AAV2/Anc80L65-CMV transduced 45.3 ± 19.7%, 48.4 ± 25.8%, and 15.1 ± 3.5% of the SCs in the apical, middle, and basal turns of the cochlea, respectively, at a titer of 2 × 10^11^ VG/ml (Table 2, Figure 3, and Supplementary Figure 4).

**FIGURE 4:**
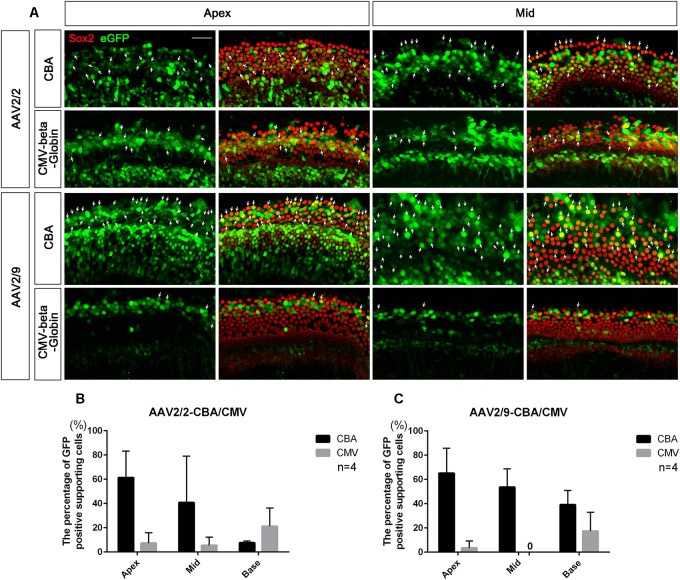
Comparison of the expression efficacy in SCs of the cochlea between AAV vectors with different promoters. **(A)** Representative confocal images of transduction of AAV2/2-CBA, AAV2/2-CMV-beta-Globin, AAV2/9-CBA and AAV2/9-CMV-beta-Globin in targeting SCs of the apical and middle turns of the cochlea *in vitro*. There were many Sox2^+^/eGFP^+^ SCs (arrows) in the apical and middle turns of the cochlea when transduced with AAV2/9-CBA. **(B)** Comparison of the expression efficiencies between AAV2/2-CBA and AAV2/2-CMV-beta-Globin in SCs of the apical, middle, and basal turns of the cochlea *in vitro* at a working titer of 1 × 10^12^ VG/ml. AAV2/2-CBA had greater expression efficacy in SCs than AAV2/2-CMV-beta-Globin on average, although there was no statistically significant difference between the expression efficiencies of AAV2/2-CBA and AAV2/2-CMV-beta-Globin in the apical, middle, or basal turn, respectively. **(C)** Comparison of the expression efficiencies between AAV2/9-CBA and AAV2/9-CMV-beta-Globin in SCs of the apical, middle, and basal turns of the cochlea *in vitro* at a working titer of 1 × 10^12^ VG/ml. AAV2/9-CBA had greater expression efficacy in SCs than AAV2/9-CMV-beta-Globin on average. *n* = 4. Scale bar: 20 μm.

### Comparison of Expression Efficacy in SCs of the Cochlea Between AAV Vectors With Different Promoters

AAV2/2-CBA had greater expression efficacy on average than AAV2/2-CMV in SCs of the whole cochlea (36.56 ± 17.70% vs. 11.33 ± 6.51%, *p* = 0.0367), while AAV2/9-CBA induced more SCs of the cochlea on average to express eGFP than AAV2/9-CMV (52.58 ± 12.02% vs. 6.97 ± 6.35%, *p* = 0.0005) at a titer of 1 × 10^12^ VG/ml (Figure 4). This indicates that the CBA promoter is more efficient than the CMV-beta-Globin promoter when driving target gene expression in SCs.

### Transduction of AAV2/2-CBA, AAV2/9-CBA, and AAV2/Anc80L65-CMV in Targeting HCs and SCs After *in vivo* Injection

**FIGURE 5:**
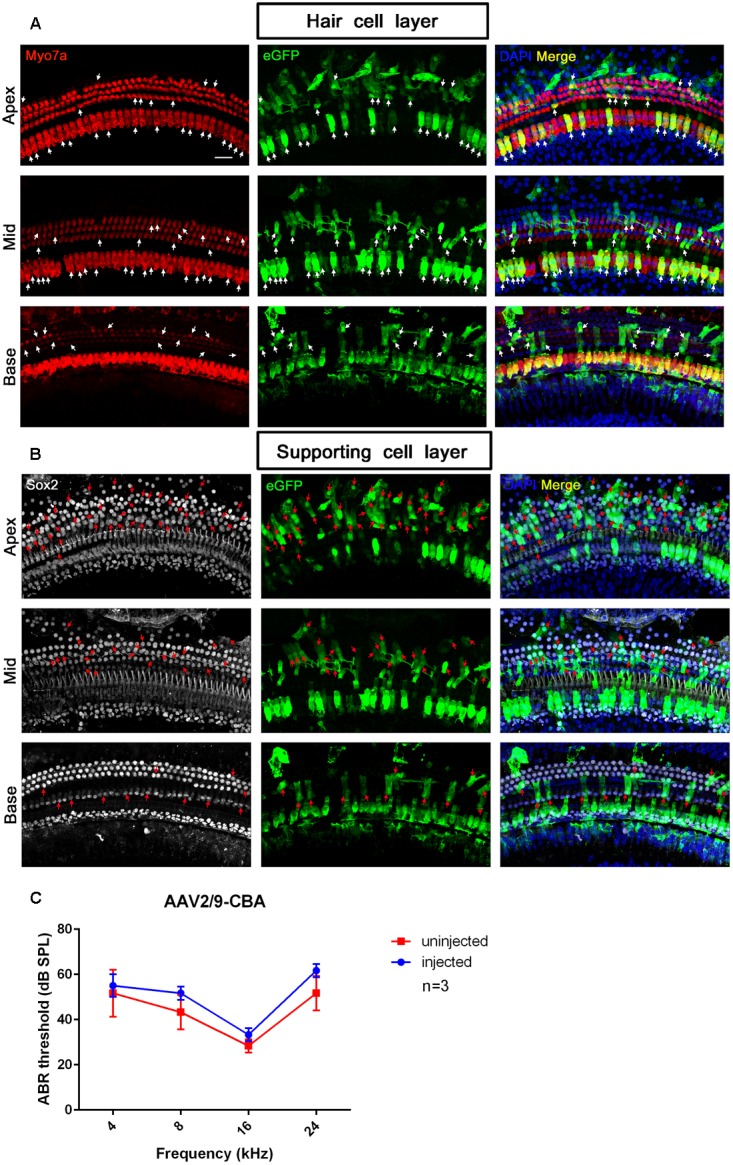
The confocal images and ABR test results of AAV2/9-CBA transduction into the cochlea after *in vivo* injection. **(A)** Transduction of AAV2/9-CBA in HCs of the apical, middle, and basal turns of the cochlea after *in vivo* injection. The white arrows indicate the Myo7a^+^/eGFP^+^ HCs. **(B)** Transduction of AAV2/9-CBA in SCs of the apical, middle, and basal turns of the cochlea after *in vivo* injection. The transduction efficiency of AAV2/9-CBA in SCs gradually decreased from the apex to the base. The tonotopic gradient with more eGFP^+^ SCs at the apex than in the base was similar to what was seen *in vitro*. The red arrows indicate the Sox2^+^/eGFP^+^ SCs. **(C)** The ABR thresholds of the injected ears were not statistically significantly different compared to the un-injected control ears 1 month after injection of AAV2/9-CBA into the inner ears (*p* > 0.05, *n* = 3). Scale bar: 20 μm.

**FIGURE 6:**
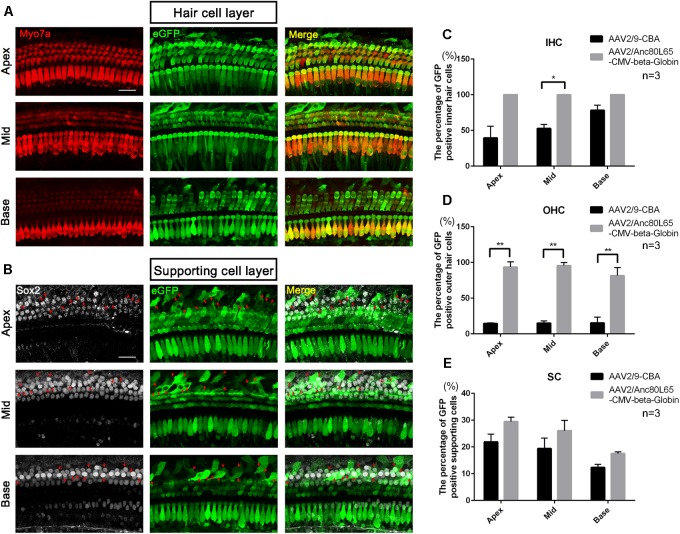
The confocal images of AAV2/Anc80L65-CMV-beta-Globin transduction into the cochlea after *in vivo* injection and comparison of the *in vivo* transduction efficiencies in IHCs, OHCs, and SCs between AAV2/9-CBA and AAV2/Anc80L65-CMV-beta-Globin. **(A)** Transduction of AAV2/Anc80L65-CMV-beta-Globin in HCs of the apical, middle, and basal turns of the cochlea after *in vivo* injection. **(B)** Transduction of AAV2/Anc80L65-CMV-beta-Globin in SCs of the apical, middle, and basal turns of the cochlea after *in vivo* injection. The red arrows indicate the Sox2^+^/eGFP^+^ SCs. **(C)** Comparison of the *in vivo* transduction efficiencies in IHCs between AAV2/9-CBA and AAV2/Anc80L65-CMV-beta-Globin. **(D)** Comparison of the *in vivo* transduction efficiencies in OHCs between AAV2/9-CBA and AAV2/Anc80L65-CMV-beta-Globin. Overall, the *in vivo* transduction efficiency of AAV2/Anc80L65-CMV-beta-Globin in IHCs and OHCs was statistically significantly greater than the transduction efficiency of AAV2/9-CBA. **(E)** Comparison of the *in vivo* transduction efficiencies in SCs between AAV2/9-CBA and AAV2/Anc80L65-CMV-beta-Globin. AAV2/Anc80L65-CMV-beta-Globin induced more SCs to express eGFP than AAV2/9-CBA on average (24.33 ± 1.83% vs. 17.87 ± 1.88%, *p* = 0.0130). ^∗^*p* < 0.0167, ^∗∗^*p* < 0.0033, *n* = 3. Scale bar: 20 μm.

Although the *in vitro* data showed that AAV2/2-CBA had good performance in terms of transduction and gene expression in HCs and SCs, the *in vivo* experiment resulted in few eGFP^+^ HCs or SCs (data not shown). One month after injection of AAV vectors into the scala media of the inner ear, the organ of Corti was isolated from the cochlea and the transduction efficiencies (the percentages of eGFP^+^ cells) in HCs and SCs of the apical, middle, and basal turns were calculated separately. For AAV2/9-CBA, the percentages of eGFP^+^ IHCs in the apical, middle, and basal turns were 39.6 ± 16.3%, 52.7 ± 5.7%, and 78.3 ± 7.2%, respectively, while the average transduction efficiency in OHCs was about 15% in all three turns (Table 5 and Figure 5). After *in vivo* injection, AAV2/Anc80L65-CMV transduced 100% of the IHCs and about 90% of the OHCs. We observed similar levels of Anc80L65 transduction in HCs from the base to the apex in the three injected mice. We also found a tonotopic gradient for the transduction of Anc80L65 in SCs *in vivo*, with more SCs expressing eGFP at the apex than in the base. The transduction efficiencies of AAV2/Anc80L65-CMV were higher than that of AAV2/9-CBA in IHCs of the middle turn and in OHCs of the three regions of the cochlea. The transduction efficiencies in SCs of the apical, middle, and basal turns were not significantly different between AAV2/Anc80L65-CMV and AAV2/9-CBA. AAV2/Anc80L65-CMV induced more SCs to express eGFP than AAV2/9-CBA on average (24.33 ± 1.83% vs. 17.87 ± 1.88%, *p* = 0.0130) (Table 5, Figure 6, and Supplementary Figure 5).

**Table 5:** Transduction efficiencies of AAV2/9-CBA and AAV2/Anc80L65-CMV-beta-Globin in IHCs, OHCs, and SCs after *in vivo* injection.

Cell type	AAV type	Apex (%)	Mid (%)	Base (%)
IHCs	AAV2/9-CBA	39.6 ± 16.3	52.7 ± 5.7	78.3 ± 7.2
	AAV2/Anc80L65-CMV-beta-Globin	100.0 ± 0.0	100.0 ± 0.0	100.0 ± 0.0
OHCs	AAV2/9-CBA	14.4 ± 0.9	15.0 ± 3.0	15.3 ± 8.1
	AAV2/Anc80L65-CMV-beta-Globin	93.5 ± 7.2	95.6 ± 4.4	81.6 ± 11.4
SCs	AAV2/9-CBA	21.9 ± 2.8	19.4 ± 4.0	12.3 ± 1.2
	AAV2/Anc80L65-CMV-beta-Globin	29.4 ± 1.7	26.1 ± 3.8	17.5 ± 0.7

*The values were represented as mean ± standard deviation (n = 3).*

### AAV2/9-CBA Transduction in the Neonatal Inner Ear Does Not Impair Hearing

One month after injection of AAV2/9-CBA into the neonatal cochlea, we performed ABR tests to compare the hearing thresholds of the injected and un-injected ears. Although the ABR threshold of the injected ears was elevated by about 3, 8, 5, and 10 dB at frequencies of 4, 8, 16, and 24 kHz, respectively, there were no statistically significant differences between the injected and un-injected ears, which showed that hearing could be preserved after AAV transduction into the inner ear in mice (Figure 5).

The exact *p*-values for all experiments were presented in Supplementary Table 2.

## Discussion

The concept of gene therapy was first proposed by Friedmann and Roblin (1972), and it was initially studied for the treatment of tumors (Chada et al., 2007), cardiovascular diseases (Muller et al., 2007), and central nervous system diseases (Ryan and Federoff, 2007). Research into the use of gene therapy in the field of otology is a recent phenomenon. By transducing exogenous genes or gene editing systems into the specific cells of the mammalian inner ear, it is possible to regulate the expression level of targeted genes in the inner ear cells for the purpose of recovering the structure and function of the auditory organ.

The key to the success of gene therapy is to choose safe and efficient vectors and appropriate pathways for gene transduction. Various viral vectors, including adenovirus (Ad) (Mondain et al., 1998; Stover et al., 2000; Dazert et al., 2001; Jero et al., 2001; Luebke et al., 2001b; Nakaizumi et al., 2004; Rejali et al., 2007; Lin et al., 2011), AAV (Lalwani et al., 1996, 1998, 2002; Kho et al., 2000; Luebke et al., 2001a; Stone et al., 2005; Bedrosian et al., 2006; Liu et al., 2007), herpes simplex virus (HSV) (Derby et al., 1999; Staecker et al., 2007), lentivirus (Han et al., 1999), and retrovirus (Zheng et al., 1998), have been applied to study gene transduction into cells of the inner ear.

Retrovirus, lentivirus, and HSV are not usually used to study gene therapy for sensorineural hearing loss because these viruses do not transduce HCs efficiently, and the expression level of the delivered genes tends to be very low (Sacheli et al., 2013). In contrast, Ad vectors have been widely applied to transduce cells of the cochlea and the vestibule (Raphael et al., 1996; Mondain et al., 1998; Stover et al., 2000; Dazert et al., 2001; Jero et al., 2001; Luebke et al., 2001b), and in mice Ad can transduce spiral ganglion cells and the epithelial layer of the scala tympani and Reissner’s membrane (Jero et al., 2001). However, although Ad is capable of transducing inner ear cells, its application in gene therapy for deafness is limited by some properties such as its inability to integrate into the host genome, its transient existence in cells, its strong immunogenicity, and its lack of tissue specificity.

Some characteristics of AAV make it a very attractive tool for transducing genes or gene editing systems (Flotte et al., 1996; Bueler, 1999; Snyder, 1999; Carter and Samulski, 2000; Peel and Klein, 2000; Rabinowitz and Samulski, 2000). AAV is a replication-defective virus containing single-stranded DNA, and it is suitable for the treatment of genetic and chronic diseases because it can transduce both mitotic and post-mitotic cells and because it has the advantages of high safety, weak immunogenicity, a wide host range, stable physical properties, and long-lasting expression. Many studies have demonstrated that AAV is the preferred vector for treating sensorineural hearing loss (Li Duan et al., 2002; Lustig and Akil, 2012). After introducing five different serotypes of AAV vectors into the cochlea of adult mice aged 2–12 months through the scala media, Kilpatrick et al. (2011) found that HCs, SCs, spiral ganglion cells, and the spiral ligament could all be transduced. Among the five serotypes (1, 2, 5, 6, and 8), AAV8 had the highest transduction rate with a higher rate in IHCs and a lower rate in SCs. Askew et al. (2015) incubated AAV-CMV-eGFP reporter vectors containing capsid serotypes 1, 2, 6, 8, or 9 with organotypic mouse cochlear cultures from P0 mice, and quantification of the viral transduction rates for the whole cochlea showed that AAV serotype 2/1 transduced the greatest number of HCs at equivalent viral titers for each serotype. AAV2/1 transduced an average of 58% of the HCs along the length of the cochlea compared to only 14% for AAV2/6, the serotype with the next highest viral transduction rate. They noted a tonotopic gradient of viral transduction, which was most apparent for AAV2/1, with more total HCs expressing eGFP in the base of the cochlea (up to 95%) than at the apex (up to 54%). The rate of viral transduction of IHCs declined sharply from the base to the apex, whereas viral transduction rates in OHCs remained higher along the entire base-to-apex axis. The mechanism behind the basal-apical gradient is not clear. In our work, there was a similar tonotopic gradient for viral transduction with the AAV2/2-CBA, AAV2/9-CBA, AAV2/2-CMV-beta-Globin, and AAV2/9-CMV-beta-Globin vectors with more eGFP^+^ OHCs in the base of the cochlea compared to the apex. However, the tonotopic gradient for viral transduction of SCs with AAV2/2-CBA *in vitro* and AAV2/Anc80L65-CMV-beta-Globin *in vivo* was different from that of HCs, and more eGFP^+^ SCs were seen at the apex than in the base of the cochlea. The transduction efficiencies of AAV vectors targeting HCs have not been completely consistent between studies, and this might be due to factors such as the serotype, the promoter, the developmental stage of the inner ear of the experimental animals, and the route of virus introduction.

Anc80L65, a unique AAV capsid, was designed to mimic an ancestral form of several AAV serotypes (1, 2, 6, 8, and 9) in order to minimize the antigenic similarity to naturally circulating AAVs that poses a problem in clinical translation of AAV gene therapies (Zinn et al., 2015). Anc80L65 was shown to be a potent gene transfer agent in liver, retina, and muscle (Zinn et al., 2015), and soon after these initial experiments, Landegger et al. (2017), Pan et al. (2017), and Suzuki et al. (2017) characterized its tropism in the inner ear *in vivo*. After systemic injection, the safety of Anc80L65 was similar in mice and non-human primates. The pre-existing immunity against circulating AAVs limits the efficacy of conventional AAV vectors, but the antigenicity of Anc80L65 is different from that of circulating AAVs, and this provides a potential benefit in terms of avoiding pre-existing immunity. The low transduction efficiency of conventional AAV vectors in OHCs has hampered the development of gene therapy for treating sensorineural hearing loss, but the highly effective transduction of Anc80L65 in OHCs overcomes this shortcoming and makes it a valuable vector for treating hereditary deafness and balance disorders. Suzuki et al. (2017) demonstrated that Anc80L65 could target almost 100% of IHCs and about half of the OHCs throughout the cochlea of adult mice after injection into the posterior semicircular canal. In our study, Anc80L65 showed consistently and qualitatively greater eGFP expression in IHCs and OHCs compared to other tested AAV vectors *in vitro* as well as *in vivo*. After *in vivo* injection into the scala media, Anc80L65 transduced 100% of the IHCs and about 90% of the OHCs. Although the transduction rate of Anc80L65 in SCs *in vivo* was about 25% on average, it still did not reach the desired level.

In addition to serotypes, promoters also play an important role in transgene expression. Most of the early studies in cochlear gene transduction relied on the human CMV promoter, but improvements in gene transduction systems have greatly increased the number of candidate promoters to choose from. The CAG promoter, which contains the sequences of both the CBA promoter and CMV promoter, shows strong and constitutive expression activity in many cell types. However, the activity of the CAG promoter is confined to cochlear HCs in mice and guinea pigs with no expression activity in SCs (Liu et al., 2005, 2007; Stone et al., 2005; Bedrosian et al., 2006). Askew et al. (2015) measured the activities of the CMV, CBA, mouse phosphoglycerate kinase 1, and synapsin 1 promoters in cochlear cultures *in vitro* using the AAV2/1 vector for delivery and eGFP expression as the readout of promoter activity. It was found that both the CMV and CBA promoters drove robust eGFP expression in HCs as well as many types of SCs in the cochlea, but there were no specific results for the transduction efficiency of AAV vectors targeting SCs in their study. In cochleae injected via the round window membrane with AAV2/1-CBA-eGFP, 59 ± 2% (*n* = 2) of the IHCs were eGFP^+^, and with AAV2/1-CMV-eGFP 70 ± 9% were eGFP^+^ (*n* = 4). This indicated that the CMV promoter led to greater expression in IHCs than the CBA promoter, which is consistent with the results of our present study.

Our study demonstrated that AAV vectors with different serotypes had different affinities for IHCs and OHCs. In our *in vitro* experiments, AAV of serotype 2 had high transduction efficiency in OHCs, while Anc80L65 could transduce a large portion of both IHCs and OHCs when injected at appropriate concentrations. The transduction efficiency of AAV2/Anc80L65-CMV approached 100% in HCs, which suggests that this is a very promising vector for inner ear gene therapy. We also found that the CMV-beta-Globin promoter had greater expression efficiency than the CBA promoter when driving transgene expression in HCs of the cochlea, whereas the CBA promoter had greater activity than the CMV-beta-Globin promoter in SCs. Thus, if the target of gene therapy is HCs, we can choose AAV vectors with the CMV promoter, but when the therapeutic target is SCs, the CBA promoter might be a better choice. In our work, the AAV vectors with the CMV-beta-Globin promoter contain a WPRE regulatory element which is thought to stabilize the mRNA, but the AAV vectors with the CBA promoter do not contain such an element. Therefore, an additional effect of the WPRE regulatory element on enhancing the eGFP expression cannot be ruled out. There is one more noteworthy issue which is the different sources of the AAV vectors with CBA promoter and CMV-beta-Globin promoter. Although the quality testing reports provided by the two facilities showed that the AAV vectors were of high quality and good stability, an effect of virus preparation from different facilities on the transduction efficiencies should be taken into consideration.

Landegger et al. (2017) used C57BL/6 or CBA/CaJ mice for *in vitro* experiments and the transduction efficiencies of AAV vectors between the two strains were not significantly different. It indicates that the transduction of AAV vectors in HCs and SCs of the cochlea would hardly be affected by the mouse strains which were used to investigate the transduction efficiencies. C57BL/6J is the most widely used inbred strain and the first to have its genome sequenced^1^. This strain is homozygous for *Cdh23^ahl^*, the age related hearing loss 1 mutation, which on this background results in progressive hearing loss with onset after 10 months of age. ICR may serve as a general purpose strain and is known for susceptibility to induced colon cancer^2^. C57BL/6J mice were previously used for *in vivo* microinjection in our research, but we found that the survival rate of the post-injection pups was low. There was no morphological or functional difference in the neonatal inner ear between C57BL/6J mice and ICR mice (data not shown). Since ICR mice have better maternity and stronger fertility than C57BL/6J mice, we used ICR mice for the *in vivo* experiments.

Injection of AAV vectors into the scala media was a safe and efficient way to transduce exogenous genes in HCs and SCs of the inner ear, but the low transduction rate of AAV vectors targeting SCs limits their application in treating sensorineural hearing loss caused by defects in SCs. In consideration of the important role of SCs in gene therapy and regenerative medicine, it is critical to find safe and efficient vectors for gene delivery into SCs. In previous studies (Liu et al., 2007; Luebke et al., 2009; Askew et al., 2015; Shu et al., 2016), there were either no detailed results of the transduction rate of AAV vectors in SCs or the transduction efficiency was very low (about 5% on average). We found that AAV2/2 and AAV2/9 with the CBA promoter had much higher expression efficiencies in SCs *in vitro*, which could reach 61.4 ± 21.9% and 65.1 ± 20.6%, respectively, at the apex of the cochlea and that AAV2/9-CBA could transduce 21.9 ± 2.8% of the SCs at the apex after *in vivo* injection. Landegger et al. (2017) found that the pattern of eGFP expression in IHCs and OHCs was similar in long-term culture with both AAV2 and Anc80L65. However, the *in vivo* results indicated that AAV2 could transduce only small numbers of IHCs and that transduction of OHCs was minimal (<5%). In contrast, Anc80L65 transduced nearly 100% of the IHCs and ∼90% of the OHCs after *in vivo* injection. This means that AAV2/2 could transduce HCs with high efficiency *in vitro*, but the transduction efficiency was sharply reduced *in vivo*. We also found a similar phenomenon for AAV2/2-CBA, which could transduce both HCs and SCs efficiently *in vitro* but had poor performance after *in vivo* injection. It is currently unclear why AAVs behave so differently when applied to explanted organs of Corti vs. *in vivo* injection. Even though several AAV vectors can transduce HCs with high efficiency, there are few AAV vectors that have good performance in transducing SCs, and the level of eGFP expression in target cells changes when using the same AAV serotype but with different promoter choice. Thus, when a new AAV vector is being constructed for the transduction of SCs, the promoter will be a very important factor to be considered in addition to the capsid. Although eGFP expression in SCs in our study was greater than previously reported, the total transduction efficiency still did not reach the desired level. In future studies, we would like to investigate the transduction efficiencies of AAV-CBA with different serotypes to determine if any serotypes of AAV-CBA can drive the expression of exogenous genes in SCs with high efficiency *in vivo*. Meanwhile, a systematic study of virus titers for *in vivo* injection will be performed in a follow-up study. All of this work will help to identify appropriate vectors for gene delivery into specific cell types of the inner ear, and such vectors will likely advance the possibility of using gene therapy to treat sensorineural hearing loss and other inner ear deficiencies.

## Author Contributions

XG was responsible for the study design and implementation, data analysis and manuscript preparation. LG, BD, WL, and RC were responsible for the data collection, statistical analysis, and manuscript preparation. YS, XH, and HL played an important role in study design and guidance and were responsible for the revision of the manuscript. All the authors approved the final version and agreed to be accountable for all aspects of the work in ensuring that questions related to the accuracy or integrity of any part of the work are appropriately investigated and resolved.

## Conflict of Interest Statement

The authors declare that the research was conducted in the absence of any commercial or financial relationships that could be construed as a potential conflict of interest.

## Footnotes

1 https://www.jax.org/strain/000664

2 https://www.jax.org/strain/009122
