# Supplementary material for: Transduction of Adeno-Associated Virus Vectors Targeting Hair Cells and Supporting Cells in the Neonatal Mouse Cochlea
Source: Front Cell Neurosci. 2019 Jan 24;13:8. doi: 10.3389/fncel.2019.00008 (PMC6353798; doi:10.3389/fncel.2019.00008)
Supplement: Supplementary file 1 [file Data_Sheet_1.docx]

*Supplementary Material*

**Transduction of Adeno-associated Virus Vectors Targeting Hair Cells and Supporting Cells in the Neonatal Mouse Cochlea**

**Xi Gu,** **Renjie Chai, Luo Guo, Biao Dong, Wenyan Li, Yilai Shu^*^, Xinsheng Huang^*^, Huawei Li^*^**

*** Correspondence:**

Dr. Yilai Shu, email: [yilai_shu@fudan.edu.cn](mailto:yilai_shu@fudan.edu.cn)

Dr. Xinsheng Huang, email: [huang.xinsheng@zs-hospital.sh.cn](mailto:huang.xinsheng@zs-hospital.sh.cn)

Dr. Huawei Li, email: [hwli@shmu.edu.cn](mailto:hwli@shmu.edu.cn)

**Supplementary Figures**

**

**

**Supplementary Figure 1. The in vitro transduction efficiency of AAV vectors with the CBA promoter in targeting HCs at different working titers** When the concentration of AAV2/2-CBA was increased, **(a, b)** the transduction efficiencies in targeting IHCs and OHCs did not increase significantly. When the concentration of AAV2/9-CBA was increased, **(c)** the percentage of eGFP^+^ IHCs increased significantly in the apical, middle, and basal turns of the cochlea; **(d)** the percentage of eGFP^+^ OHCs increased significantly in the apical and middle turns of the cochlea. * *p* < 0.0167, ** *p* < 0.0033, n = 5.





**Supplementary Figure 2. The in vitro transduction efficiency of AAV vectors with the CMV-beta-Globin promoter in targeting HCs at different working titers** When the working titer was increased from 0.5 × 10^11^ VG/ml to 1 × 10^11^ VG/ml, **(a)** the percentage of eGFP^+^ IHCs in the basal turn transduced by AAV2/2-CMV increased significantly; **(b)** the percentage of eGFP^+^ OHCs transduced by AAV2/2-CMV did not increase significantly; the transduction efficiencies of AAV2/9-CMV in **(c)** IHCs and **(d)** OHCs did not increase significantly. **(e, f)** When the working titer was 2 × 10^11^ VG/ml, the transduction efficiencies of AAV2/Anc80L65-CMV in IHCs and OHCs were very close to 100%. Viral transduction rates of AAV2/Anc80L65-CMV in IHCs and OHCs were consistently high along the base-to-apex axis. The blue asterisks (**) represent the results of statistical analysis of comparing the transduction efficiencies between the working titers of 1 × 10^11^ VG/ml and 0.5 × 10^11^ VG/ml. The red asterisks (*/**) represent the results of statistical analysis of comparing the transduction efficiencies between the working titers of 2 × 10^11^ VG/ml and 1 × 10^11^ VG/ml. * *p* < 0.0167, ** *p* < 0.0033, n = 5.


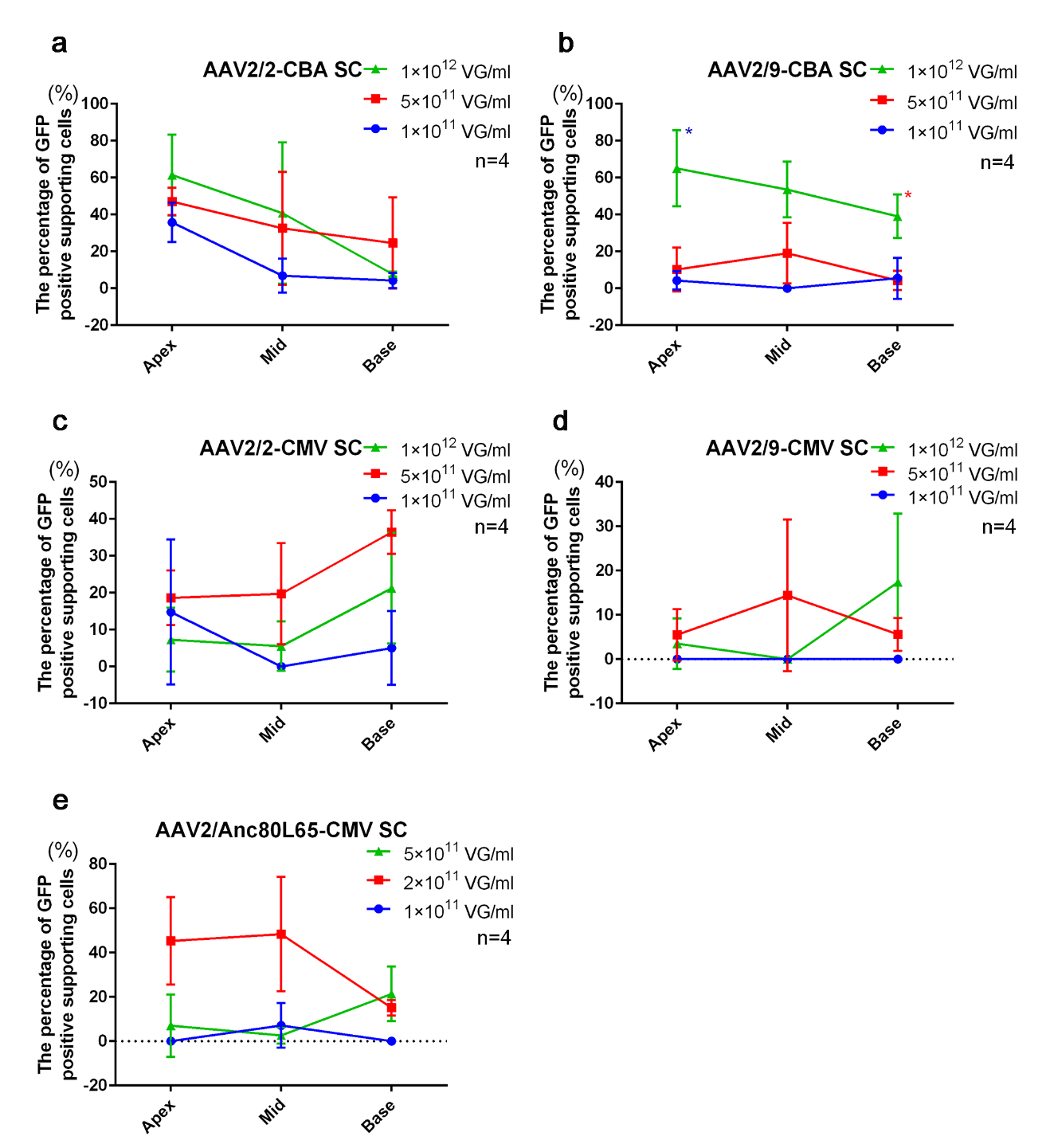


**Supplementary Figure 3. The in vitro transduction efficiency of AAV vectors in SCs at different working titers** The percentages of eGFP^+^ SCs in the apical, middle, and basal turns of the cochlea with different working titers were showed respectively when transduced by AAV2/2-CBA **(a)**, AAV2/9-CBA **(b)**, AAV2/2-CMV **(c)**, AAV2/9-CMV **(d)**, and AAV2/Anc80L65-CMV **(e)**. **(a, b)** The blue asterisk (*) represents the result of statistical analysis of comparing the transduction efficiencies between the working titers of 1 × 10^12^ VG/ml and 1 × 10^11^ VG/ml. The red asterisk (*) represents the result of statistical analysis of comparing the transduction efficiencies between the working titers of 1 × 10^12^ VG/ml and 5 × 10^11^ VG/ml. **(c, d)** When the working titer was increased, the transduction efficiencies of AAV2/2-CMV and AAV2/9-CMV in SCs of the apical, middle, and basal turns did not increase statistically significantly. **(e)** When compared in the three regions of the cochlea respectively, the transduction efficiencies of AAV2/Anc80L65-CMV in SCs did not increase statistically significantly with the working titer increased. The average transduction efficiencies of AAV2/Anc80L65-CMV in SCs of the whole cochlea increased significantly (2.35 ± 3.36% vs. 36.24 ± 13.00%, *p* = 0.0023) with the working titer increased from 1 × 10^11^ VG/ml to 2 × 10^11^ VG/ml. * *p* < 0.0167, n = 4.


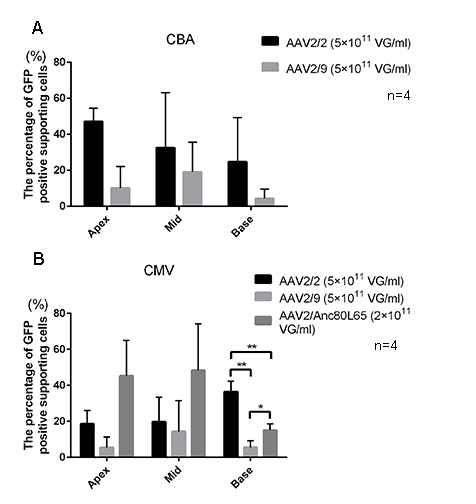


**Supplementary Figure 4. Comparison of the in vitro transduction efficiencies in SCs of the cochlea between different serotypes of AAV vectors with the same promoters** **(A)** Comparison of transduction efficiencies between AAV2/2-CBA and AAV2/9-CBA in SCs at a titer of 5 × 10^11^ VG/ml. There was no significant difference between the transduction efficiencies of AAV2/2-CBA and AAV2/9-CBA in SCs of the apical, middle, and basal turns of the cochlea. **(B)** When the titer of AAV2/2 and AAV2/9-CMV-beta-Globin was 5 × 10^11^ VG/ml and the titer of AAV2/Anc80L65-CMV-beta-Globin was 2 × 10^11^ VG/ml, comparison of the transduction efficiencies among the three AAV vectors in SCs at different regions of the cochlea. * *p* < 0.0167, ** *p* < 0.0033, n = 4.

**

**

**Supplementary Figure 5. Comparison of the transduction efficiencies in OHCs or SCs among three regions (apical, middle, and basal turns) of the cochlea when transduced by (A, E)** AAV2/2-CBA in vitro (1 × 10^11^ VG/ml), **(B)** AAV2/9-CBA in vitro (1 × 10^11^ VG/ml), **(C)** AAV2/2-CMV in vitro (0.5 × 10^11^ VG/ml), **(D)** AAV2/9-CMV in vitro (1 × 10^11^ VG/ml), **(F)** AAV2/Anc80L65-CMV-beta-Globin in vivo. * *p* < 0.05, ** *p* < 0.01.

**Supplementary Tables**

**Supplementary Table 1 The transduction efficiencies of AAV2/2-CBA, AAV2/9-CBA, AAV2/2-CMV, AAV2/9-CMV, and AAV2/Anc80L65-CMV in IHCs and OHCs of the apical, middle, and basal turns of the cochlea with two different lots at the working titer of 1 × 10^11^ VG/ml. The values were represented as mean ± standard deviation. (n = 5)**

| AAV type | Cell type | Lots | Apex (%) | Mid (%) | Base (%) |
| --- | --- | --- | --- | --- | --- |
| AAV2/2-CBA | IHCs | Lot Ⅰ | 6.0 ± 3.2 | 3.9 ± 2.5 | 11.7 ± 12.7 |
|  |  | Lot Ⅱ | 5.8 ± 2.5 | 4.8 ± 3.3 | 12.4 ± 11.9 |
|  | OHCs | Lot Ⅰ | 35.1 ± 3.3 | 47.8 ± 16.4 | 68.2 ± 17.7 |
|  |  | Lot Ⅱ | 36.6 ± 1.8 | 43.6 ± 8.7 | 67.6 ± 16.2 |
| AAV2/9-CBA | IHCs | Lot Ⅰ | 6.9 ± 2.9 | 4.2 ± 0.9 | 6.6 ± 1.7 |
|  |  | Lot Ⅱ | 7.9 ± 1.2 | 6.0 ± 2.30 | 7.6 ± 1.2 |
|  | OHCs | Lot Ⅰ | 4.5 ± 1.1 | 3.0 ± 1.9 | 26.2 ± 5.9 |
|  |  | Lot Ⅱ | 4.9 ± 0.9 | 2.9 ± 1.7 | 25.2 ± 6.4 |
| AAV2/2-CMV | IHCs | Lot Ⅰ | 35.9 ± 24.3 | 45.5 ± 18.8 | 67.0 ± 6.5 |
|  |  | Lot Ⅱ | 34.4 ± 20.7 | 49.4 ± 16.7 | 68.4 ± 6.7 |
|  | OHCs | Lot Ⅰ | 68.3 ± 19.8 | 63.8 ± 22.8 | 76.0 ± 21.6 |
|  |  | Lot Ⅱ | 70.4 ± 17.3 | 74.0 ± 15.8 | 75.4 ± 20.6 |
| AAV2/9-CMV | IHCs | Lot Ⅰ | 47.9 ± 19.2 | 58.8 ± 20.0 | 73.6 ± 6.5 |
|  |  | Lot Ⅱ | 48.0 ± 18.9 | 59.8 ± 16.4 | 73.4 ± 8.3 |
|  | OHCs | Lot Ⅰ | 12.1 ± 11.7 | 30.1 ± 12.6 | 53.5 ± 28.1 |
|  |  | Lot Ⅱ | 12.6 ± 10.7 | 36.5 ± 19.5 | 54.7 ± 25.6 |
| AAV2/Anc80L65-CMV | IHCs | Lot Ⅰ | 76.7 ± 11.8 | 82.5 ± 6.4 | 83.8 ± 8.8 |
|  |  | Lot Ⅱ | 78.6 ± 13.8 | 80.9 ± 7.7 | 82.2 ± 7.1 |
|  | OHCs | Lot Ⅰ | 68.9 ± 8.4 | 75.8 ± 6.6 | 81.6 ± 8.0 |
|  |  | Lot Ⅱ | 59.7 ± 15.1 | 74.2 ± 7.9 | 79.7 ± 6.0 |

**Supplementary Table 2 The *p*-values for each comparison of the experiments**

| Pairs of comparison | | *p* values | | |
| --- | --- | --- | --- | --- |
|  |  | Apex | Mid | Base |
| Transduction efficiency of AAV2/2-CBA in IHCs | 1 × 10^11^ VG/ml vs. 1 × 10^12^ VG/ml | 0.6875 | 0.3301 | 0.1032 |
| Transduction efficiency of AAV2/2-CBA in OHCs | 1 × 10^11^ VG/ml vs. 1 × 10^12^ VG/ml | 0.0586 | 0.2760 | 0.4603 |
| Transduction efficiency of AAV2/9-CBA in IHCs | 1 × 10^11^ VG/ml vs. 1 × 10^12^ VG/ml | 0.0079 | 0.0015 | 0.0070 |
| Transduction efficiency of AAV2/9-CBA in OHCs | 1 × 10^11^ VG/ml vs. 1 × 10^12^ VG/ml | 0.0021 | 0.0067 | 0.0556 |
| Transduction efficiency of AAV2/2-CMV in IHCs | 0.5 × 10^11^ VG/ml vs. 1 × 10^11^ VG/ml | 0.1979 | 0.0364 | 0.0096 |
| Transduction efficiency of AAV2/2-CMV in OHCs | 0.5 × 10^11^ VG/ml vs. 1 × 10^11^ VG/ml | 0.2832 | 0.8731 | 0.6266 |
| Transduction efficiency of AAV2/9-CMV in IHCs | 0.5 × 10^11^ VG/ml vs. 1 × 10^11^ VG/ml | 0.7476 | 0.1533 | 0.0849 |
| Transduction efficiency of AAV2/9-CMV in OHCs | 0.5 × 10^11^ VG/ml vs. 1 × 10^11^ VG/ml | 0.0952 | 0.0556 | 0.0952 |
| Transduction efficiency of AAV2/Anc80L65-CMV in IHCs | 0.5 × 10^11^ VG/ml vs. 1 × 10^11^ VG/ml | 0.0007 | <0.0001 | <0.0001 |
|  | 0.5 × 10^11^ VG/ml vs. 2 × 10^11^ VG/ml | 0.0079 | 0.0079 | 0.0079 |
|  | 1 × 10^11^ VG/ml vs. 2 × 10^11^ VG/ml | 0.0079 | 0.0079 | 0.0238 |
| Transduction efficiency of AAV2/Anc80L65-CMV in OHCs | 0.5 × 10^11^ VG/ml vs. 1 × 10^11^ VG/ml | 0.0011 | 0.0003 | 0.0002 |
|  | 0.5 × 10^11^ VG/ml vs. 2 × 10^11^ VG/ml | 0.0002 | 0.0003 | 0.0084 |
|  | 1 × 10^11^ VG/ml vs. 2 × 10^11^ VG/ml | 0.0011 | 0.0023 | 0.0002 |
| Transduction efficiency of AAV2/2-CBA in SCs | 1 × 10^11^ VG/ml vs. 5 × 10^11^ VG/ml | 0.1372 | 0.1928 | 0.1975 |
|  | 1 × 10^11^ VG/ml vs. 1 × 10^12^ VG/ml | 0.2000 | 0.1736 | 0.2056 |
|  | 5 × 10^11^ VG/ml vs. 1 × 10^12^ VG/ml | 0.3429 | 0.7461 | 0.2621 |
| Transduction efficiency of AAV2/9-CBA in SCs | 1 × 10^11^ VG/ml vs. 5 × 10^11^ VG/ml | 0.6571 | 0.1024 | 0.4857 |
|  | 1 × 10^11^ VG/ml vs. 1 × 10^12^ VG/ml | 0.0077 | 0.0286 | 0.0286 |
|  | 5 × 10^11^ VG/ml vs. 1 × 10^12^ VG/ml | 0.0286 | 0.0286 | 0.0053 |
| Transduction efficiency of AAV2/2-CMV in SCs | 1 × 10^11^ VG/ml vs. 5 × 10^11^ VG/ml | 0.8000 | 0.0286 | 0.0286 |
|  | 1 × 10^11^ VG/ml vs. 1 × 10^12^ VG/ml | 0.5226 | 0.4286 | 0.1429 |
|  | 5 × 10^11^ VG/ml vs. 1 × 10^12^ VG/ml | 0.1714 | 0.1306 | 0.1335 |
| Transduction efficiency of AAV2/9-CMV in SCs | 1 × 10^11^ VG/ml vs. 5 × 10^11^ VG/ml | 0.1429 | 0.1429 | 0.0286 |
|  | 1 × 10^11^ VG/ml vs. 1 × 10^12^ VG/ml | 0.4286 | >0.9999 | 0.1429 |
|  | 5 × 10^11^ VG/ml vs. 1 × 10^12^ VG/ml | 0.6571 | 0.1429 | 0.2251 |
| Transduction efficiency of AAV2/Anc80L65-CMV in SCs | 1 × 10^11^ VG/ml vs. 2 × 10^11^ VG/ml | 0.0286 | 0.0420 | 0.0286 |
|  | 1 × 10^11^ VG/ml vs. 5 × 10^11^ VG/ml | >0.9999 | 0.4540 | 0.0286 |
|  | 2 × 10^11^ VG/ml vs. 5 × 10^11^ VG/ml | 0.0571 | 0.0367 | 0.3844 |
| Transduction efficiency of AAV-CBA in IHCs | AAV2/2 vs. AAV2/9 | 0.6586 | 0.7788 | 0.4269 |
| Transduction efficiency of AAV-CBA in OHCs | AAV2/2 vs. AAV2/9 | <0.0001 | 0.0034 | 0.0043 |
| Transduction efficiency of AAV-CMV in IHCs | AAV2/2 vs. AAV2/9 | 0.4150 | 0.3111 | 0.1476 |
|  | AAV2/2 vs. AAV2/Anc80L65 | 0.0160 | 0.0092 | 0.0097 |
|  | AAV2/9 vs. AAV2/Anc80L65 | 0.0261 | 0.0552 | 0.0705 |
| Transduction efficiency of AAV-CMV in OHCs | AAV2/2 vs. AAV2/9 | 0.0012 | 0.0266 | 0.1975 |
|  | AAV2/2 vs. AAV2/Anc80L65 | 0.9526 | 0.3157 | 0.6081 |
|  | AAV2/9 vs. AAV2/Anc80L65 | <0.0001 | 0.0004 | 0.0887 |
| Expression efficiency of AAV in IHCs | AAV2/2-CBA vs. AAV2/2-CMV | 0.0359 | 0.0033 | 0.0079 |
|  | AAV2/9-CBA vs. AAV2/9-CMV | 0.0089 | 0.0016 | <0.0001 |
| Expression efficiency of AAV in OHCs | AAV2/2-CBA vs. AAV2/2-CMV | 0.0116 | 0.0087 | 0.4206 |
|  | AAV2/9-CBA vs. AAV2/9-CMV | 0.1796 | 0.0179 | 0.0600 |
| Transduction efficiency of AAV-CBA in SCs | AAV2/2 vs. AAV2/9 | 0.0286 | 0.4784 | 0.1997 |
| Transduction efficiency of AAV-CMV in SCs | AAV2/2 vs. AAV2/9 | 0.0286 | 0.6494 | 0.0003 |
|  | AAV2/2 vs. AAV2/Anc80L65 | 0.0571 | 0.1119 | 0.0016 |
|  | AAV2/9 vs. AAV2/Anc80L65 | 0.0228 | 0.0775 | 0.0099 |
| Expression efficiency of AAV in SCs | AAV2/2-CBA vs. AAV2/2-CMV | 0.0286 | 0.4850 | 0.4117 |
|  | AAV2/9-CBA vs. AAV2/9-CMV | 0.0286 | 0.0286 | 0.0709 |
| Transduction efficiency of AAV in IHCs in vivo | AAV2/9-CBA vs. AAV2/Anc80L65-CMV | 0.0233 | 0.0048 | 0.0344 |
| Transduction efficiency of AAV in OHCs in vivo | AAV2/9-CBA vs. AAV2/Anc80L65-CMV | 0.0025 | <0.0001 | 0.0018 |
| Transduction efficiency of AAV in SCs in vivo | AAV2/9-CBA vs. AAV2/Anc80L65-CMV | 0.0251 | 0.1026 | 0.1000 |
